# Effect of ionizing radiation on human skeletal muscle precursor cells

**DOI:** 10.2478/raon-2013-0058

**Published:** 2013-10-08

**Authors:** Mihaela Jurdana, Maja Cemazar, Katarina Pegan, Tomaz Mars

**Affiliations:** 1 University of Primorska, Faculty of Health Science, Izola, Slovenia; 2 Institute of Oncology Ljubljana, Department of Experimental Oncology, Ljubljana, Slovenia; 3 University of Ljubljana, Faculty of Medicine, Institute of Pathophysiology, Ljubljana, Slovenia; 4 Jožef Stefan Institute, Department of Biochemistry, Molecular and Structural Biology, Ljubljana, Slovenia

**Keywords:** myoblasts, irradiation, proliferation, interleukin 6, muscle regeneration, apoptosis

## Abstract

**Background:**

Long term effects of different doses of ionizing radiation on human skeletal muscle myoblast proliferation, cytokine signalling and stress response capacity were studied in primary cell cultures.

**Materials and methods:**

Human skeletal muscle myoblasts obtained from muscle biopsies were cultured and irradiated with a Darpac 2000 X-ray unit at doses of 4, 6 and 8 Gy. Acute effects of radiation were studied by interleukin – 6 (IL-6) release and stress response detected by the heat shock protein (HSP) level, while long term effects were followed by proliferation capacity and cell death.

**Results:**

Compared with non-irradiated control and cells treated with inhibitor of cell proliferation Ara C, myoblast proliferation decreased 72 h post-irradiation, this effect was more pronounced with increasing doses. Post-irradiation myoblast survival determined by measurement of released LDH enzyme activity revealed increased activity after exposure to irradiation. The acute response of myoblasts to lower doses of irradiation (4 and 6 Gy) was decreased secretion of constitutive IL-6. Higher doses of irradiation triggered a stress response in myoblasts, determined by increased levels of stress markers (HSPs 27 and 70).

**Conclusions:**

Our results show that myoblasts are sensitive to irradiation in terms of their proliferation capacity and capacity to secret IL-6. Since myoblast proliferation and differentiation are a key stage in muscle regeneration, this effect of irradiation needs to be taken in account, particularly in certain clinical conditions.

## Introduction

Maintenance of skeletal muscle mass and function depends on the process of muscle regeneration and the ability of satellite cells to activate into proliferative myoblasts, key cells responsible for muscle lesion repair and reconstruction.^1^ Myokine interleukin 6 (IL-6) is released from activated myoblasts in response to injury and IL-6 is an important factor promoting myoblast proliferation and differentiation during muscle regeneration.^2^–^5^ Several studies have investigated radiation effects on skeletal muscle, demonstrating that skeletal muscle damage after irradiation remains for many years. Adult skeletal muscle is considered to be radiation resistant, unless higher doses of radiation are applied.^6^–^11^ However, radiation directly inhibits muscle regeneration by damaging satellite cells, which can lead to mitotic failure and cell death.^12^ Impaired muscle regeneration following irradiation may thus be due to an insufficient number of activated satellite cells needed for fusion and repair of damaged muscle fibre. The lack of adequate muscle regeneration may also be due to impaired cytokine signalling and, finally, differentiation.^13^ This indicates that skeletal muscle is sensitive to ionizing radiation during development, that is why radiotherapy in childhood may induce muscular atrophy, a fact that is attributed to the large number of radiosensitive satellite cells during a child’s growth period.^14^ The underlying mechanism for radiation induced muscular atrophy has been insufficiently studied, so the main aim of this study was to evaluate the radiation dose-dependent effect on precursors of muscle regeneration, primary mononucleated human myoblasts, which are key cells involved in the development of adult muscle fibre and in the process of muscle regeneration. Two types of irradiation effects on human myoblast *in vitro* were studied: acute effects (determined 24 h post-irradiation) were followed by monitoring IL-6 secretion and stress response detected by the level of HSPs; long term effects (evaluated 72 h post-irradiation) were followed through proliferation capacity and cell death.

## Materials and methods

### Study design

The study was conducted at the Institute of Pathophysiology of the University of Ljubljana, where myoblast cell cultures were prepared and analysed, and at the Institute of Oncology Ljubljana, where cell cultures were exposed to ionizing radiation under controlled conditions. The experiments were approved by the Ethical Commission of the Ministry of Health of the Republic of Slovenia (permit numbers 63/01/99 and 71/05/12) and performed in compliance with the Helsinki Declaration and Good Laboratory Practice regulations. Experiments were performed on primary cultures of human myoblast that were prepared from three different donors (see below). Each primary culture was considered as independent experiment and all treatments of cells were performed at least in triplicate.

### Myoblast cultures

Experiments were performed on cultures of human myoblasts prepared as previously described.^5^,^15^–^16^ Human myoblasts were derived from satellite cells obtained from muscle tissue routinely discarded during orthopaedic operations on donor patients aged 5, 11 and 20 years, without diagnosed muscular disease. After muscle tissue cleaning and trypsinization, released muscle satellite cells were grown in advanced Minimum Essential Medium (aMEM, Invitrogen, Grand Island, NY, USA) supplemented with 10% fetal bovine serum (FBS, Invitrogen) at saturated humidity in a mixture of 5% CO_2_-enriched air at 37°C. Myoblast colonies identified by morphological characteristics and devoid of fibroblast contamination were trypsinized and further expanded. Cells were plated in six-well dishes and grown for 3 days in aMEM supplemented with 10% FBS prior to the experiments.

### Irradiation

Cells (1×10^6^ cells/ml aMEM) were plated in six-well dishes and irradiated with a dose rate of 2 Gy/min, with graded doses (2–8 Gy). A Darpac 2000 X-ray unit (Gulmay Medical Ltd, Shepperton, UK), operated at 220 kV, 10 mA, using 0.55 mm Cu filtration and 1.8 mm Al filtration was used for irradiation.

### BrdU proliferation assay

The proliferation of cells was determined by BrdU Cell Proliferation Assay, Calbiochem, Merk, Darmstadt, Germany. Cells were plated on 96-well plates (1500 cells per well in 100 μl MEM), the next day they were irradiated and the proliferation test was performed 72 h later. 10 μM arabunifuranosyl cytidine (Ara C), an inhibitor of DNA synthesis, was added to the cells to produce the negative proliferation control. BrdU was allowed to incubate with cells for 18 h. BrdU labelled cells were visualized by Anti-BrdU Antibody diluted 1:100 (supplied with the kit). The diluted Peroxidase Goat Anti-Mouse IgG HRP Conjugate was filtered through 0.2 micron filter according to instructions. Fifteen minutes after adding the Substrate Solution absorbance was measured at 450 nm and 540 nm (Victor 3 plate reader form PerkinElmer, Shelton, CT, USA).

### Assessment of apoptosis and necrosis

Cells were plated in white 24-well plates (10^5^ cells per well in 500 μl MEM) (Visiplate-24 TC, PerkinElmer, Shelton, CT, USA). After irradiation, the cells were treated for caspase detection and the culture media were collected and used to assess the release of lactate dehydrogenase (LDH, EC 1.1.1.27).

Apoptotic initiator caspase 9 (LEHD-ase) and executor caspase 3/7 (DEVD-ase) activity were measured using CaspaseGlo 9 Assay and CaspaseGlo 3/7 Assay Kits from Promega (Madison, WI, USA).

The level of necrotic cell death after cell irradiation was determined by measuring the activity of LDH in the cell culture media using a Cytotoxicity Detection Kit PLUS (Roche Diagnostics GmbH, Mannheim, Germany). Luminescence and absorbance (490 nm) were measured on a Victor 3 plate reader (PerkinElmer, Shelton, CT, USA) immediately after irradiation, 36 h and 72 h later.

### IL-6 secretion analysis

Levels of secreted IL-6 in the culture supernatant collected from the cultures 24-h after exposure to irradiation were measured using an Enzyme-Linked ImmunoSorbent Assay (ELISA) kit (Endogen, Rockford, USA), according to the manufacturer’s instructions and as described previously.^17^ IL-6 levels were calculated from concentrations of IL-6 measured in supernatants in each well.

### Western Blot analysis for HSP 27 and HSP 70

Western blot analysis was performed as described in Katalinic *et al*.^16^ from myoblast samples collected from the cultures 24-h after exposure to irradiation. In brief, myoblasts were washed in ice-cold PBS and lyzed. Protein content was determined in supernatants by the Bradford protein assay (Thermo Scientific Pierce, USARockford, IL). Homogenate samples were mixed with standard Laemmli buffer (3:1) (at 56°C, 20 min) and separated in 10% NuPage Novex Bis-Tris Gel (Invitrogen) by using the XCell SureLock electrophoresis system (Invitrogen) and transferred to a PDVF membrane (Millipore, Billerica, MA, USA). Membranes with transferred samples were blocked in a blocking buffer (0.2% (w/v) I-Block (Applied Biosystems), 0.3% (v/v) Tween 20 (Sigma-Aldrich) prepared in phosphate buffered saline (PBS)) and incubated with selected primary antibodies against HSP 27 (rabbit polyclonal antibody PA1-516, ABR-Affinity BioReagents, USARockford, IL; diluted 1:200), HSP 70 (rabbit polyclonal antibody PA3-514, ABR-Affinity BioReagents; diluted 1:200) overnight at 4°C. Next day, the membranes were incubated with alkaline phosphatase-conjugated secondary antibodies (Sigma-Aldrich). Blots were developed in 2% (v/v) NBT/BCIP (Roche, Mannheim, Germany) solution prepared in developing buffer. Membrane quantification was performed with the Chemi Genius BioImaging System (Syngen, Cambridge, UK).

### Statistical analysis

Results are presented as mean ± SD. The differences among experimental groups were calculated using one-way ANOVA, followed by Bonferroni’s post hoc test for multiple comparisons. SPSS 15.0 for Windows (SPSS, Chicago, IL, USA) was used for data analysis.

## Results

### Myoblast proliferation

**FIGURE 1.**
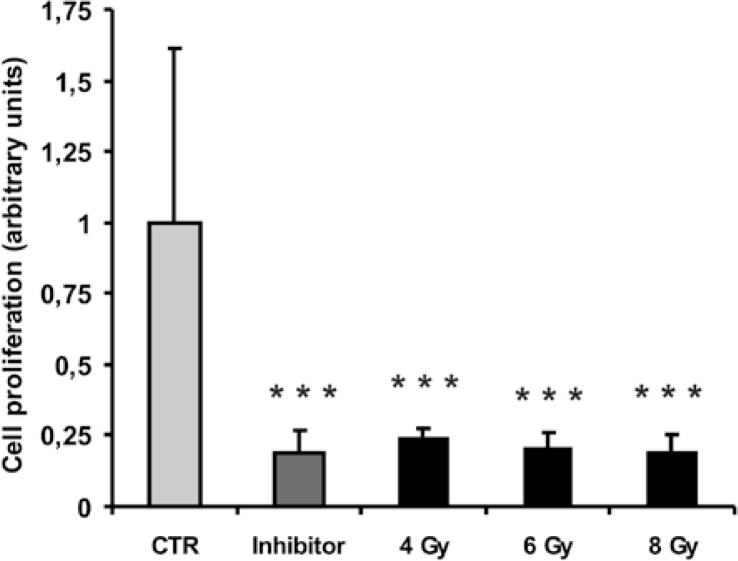
The effect of selected therapeutic doses of ionizing radiation (4 Gy, 6 Gy, 8 Gy) on the proliferation of human skeletal myoblasts assessed 72 h after irradiation. Columns and bars represent means ± SD (n= 8). Means are expressed as arbitrary units which are relative units of absorbance measurement at dual wavelengths of 450–540 nm. The control was used as the predetermined reference measurement. Statistically significant differences (p < 0.001) are indicated by ***. Control (CTR) – non-irradiated myoblast; Inhibitor – myoblasts treated with 10 μM AraC, an inhibitor of cell proliferation; 4 Gy – myoblasts irradiated with 4 Gy; 6 Gy - myoblasts irradiated with 6 Gy; 8 Gy – myoblasts irradiated with 8 Gy.

Therapeutic doses of ionizing radiation used in the experiment effectively inhibited human myoblast proliferation. Irradiation at all doses statistically significantly inhibited myoblast proliferation to the same level (∼ 0.2 of the untreated control) as the inhibitor of DNA synthesis Ara C (Figure 1).

### Assessment of cell death

**FIGURE 2.**
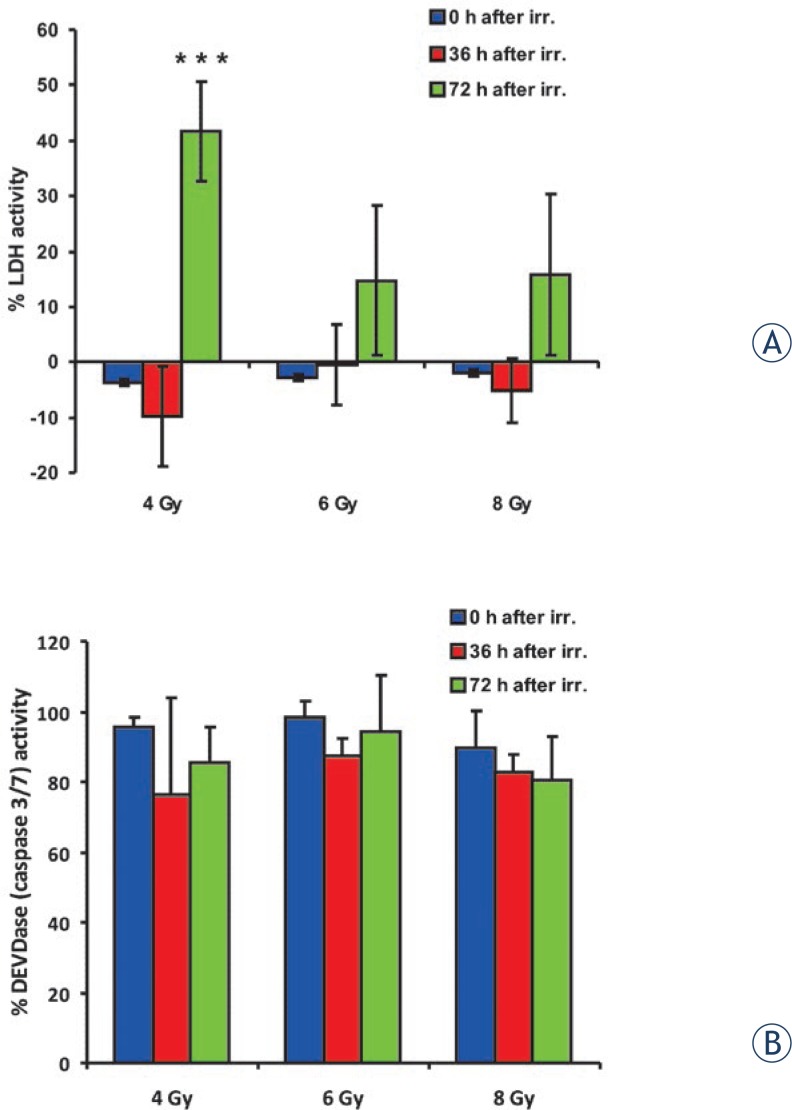
The effect of selected therapeutic doses of ionizing radiation (4 Gy, 6 Gy, 8 Gy) on cell death immediately after irradiation (0 h after IR.), 36 h after irradiation (36 h after IR.) and 72 h after irradiation (72 h after IR). Columns and bars represent means ± SD (n=4). (A) The assessment of myoblasts membranes integrity at different time points after irradiation is shown as the activity of lactate dehydrogenase (LDH) released in the medium. Statistically significant differences (p ≤ 0,001) are indicated by ***. (B) The cleavage activity of the amino acid sequence DEVD (recognised and cleaved by apoptotic caspase 3 and 7) is shown at different time points after irradiation. There were no statistically significant differences among groups.

Irradiation of myoblasts did not cause necrotic cell death, measured by LDH activity, up to 36 h after treatment at all tested doses (Figure 2A). However, a statistically significant increase in necrotic cell death occurred 72 h after irradiation with 4 Gy. LDH activity in the media at this time point increased to 42 ± 9%. Furthermore, a similar pattern was seen after irradiation with higher doses, although the increase in LDH activity was not statistically significant compared to control untreated cells.

Apoptosis of cells was measured by activation of caspases 3, 7 and 9. Irradiation of myoblasts did not result in either increased activity of initiator caspase 9 (data not shown) or activation of execution caspases 3 and 7 (Figure 2 B).

### Effects of irradiation on the secretion of IL-6

**FIGURE 3.**
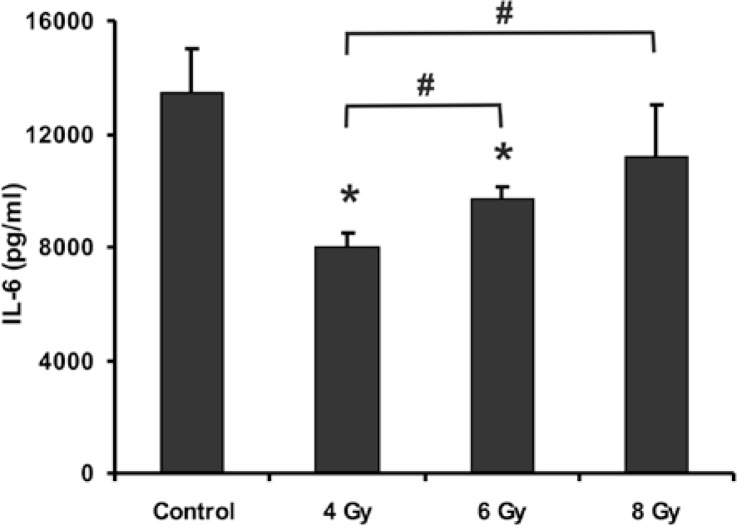
Effects of different doses of ionizing radiation on constitutive level of IL-6. Level of constitutive IL-6 secretion was estimated by ELISA in control myoblast cultures and compared with level of IL-6 secretion in myoblast cultures 24 hours after exposure to 4 Gy, 6 Gy and 8 Gy dose of irradition. Data are means ± SD (n = 18 in each independent experiment). * p < 0.05 (t-test) denotes difference in level of IL-6 in exposed cultures vs. respective level of control cultures, # significant difference between groups indicated p < 0.05 (t-test).

Levels of IL-6 were measured in supernatants of cultures 24-hours after exposure to different doses of irradiation. In control cultures that were kept in the same conditions as other cultures and were not exposed to irradiation, the constitutive level of IL-6 was 13,448 pg/ml (n=18). Exposure of myoblasts to irradiation resulted in a decreased level of IL-6 secretion in a dose dependent manner, being most pronounced in myoblasts irradiated with 4 Gy in comparison with myoblast irradiated with 6 Gy (p<0.05) or with 8 Gy (p<0.05). Irradiation of cells with 6 Gy also resulted in a statistically significant decrease of IL-6 secretion, while irradiation of myoblasts with the highest tested dose (8 Gy) did not result in decreased secretion of IL-6 (Figure 3).

### Stress protein response in myoblast exposed to irradiation

**FIGURE 4.**
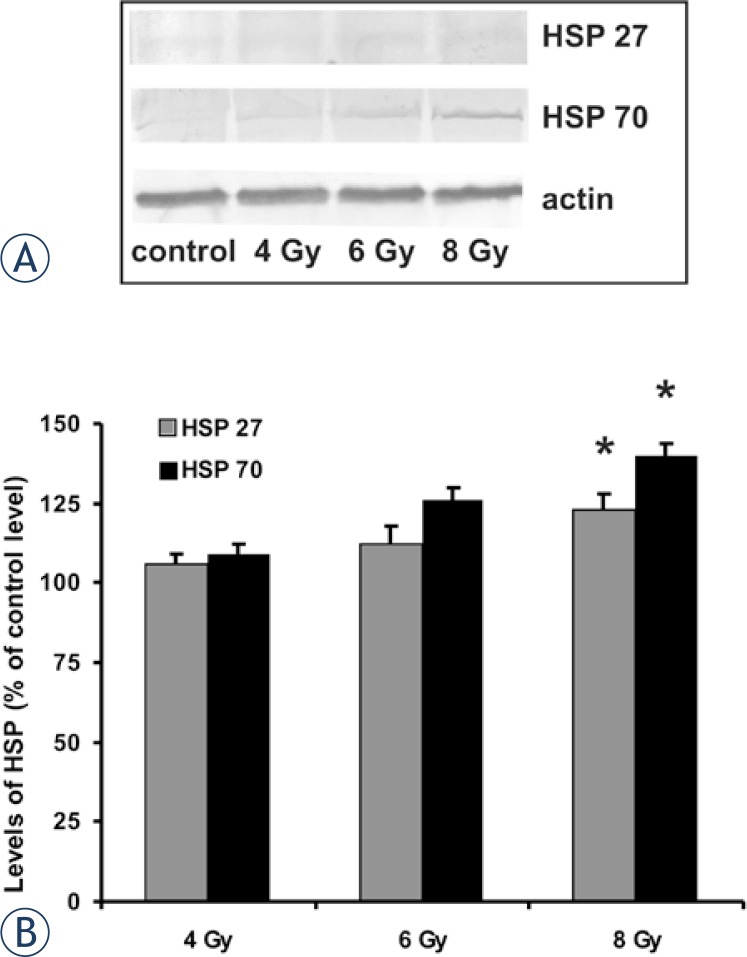
The effect of different doses of ionizing radiation on the HSP 27 and HSP 70 level in myoblasts 24 h after exposure. Representative Western blots for HSP 27 and HSP 70 (A). Relative levels of HSP 27 and HSP 70 shown as % of control level of proteins in myoblasts not exposed to irradiation (B), * p < 0.05 denotes significant difference in level of HSP in exposed myoblasts vs. level in control non-exposed myoblasts.

The two most prominent stress proteins, HSP 27 and HSP 70, were followed in myoblasts 24-hours after irradiation. Levels of both proteins were increased in myoblasts exposed to irradiation in comparison with levels in control non-exposed myoblasts, although this increase was insignificant in myoblasts exposed to lower doses of irradiation (4 Gy and 6 Gy), while in myoblasts exposed to 8 Gy irradiation, the HSP 70 level was statistically significantly increased (p < 0.05) (Figure. 4).

## Discussion

Radiation is thought to prevent mitosis of satellite cells, the primary stem cells in adult skeletal muscle, responsible for postnatal muscle growth and hypertrophy, causing breaks in strands of the cell’s DNA and in this way inhibiting muscle regeneration.^12^ Human muscle precursor myoblasts derived from satellite cells are responsible for muscle growth and regeneration.^18^,^19^ Radiation is well-known to affect muscle cells during development by impairing their activation, proliferation and differentiation.^20^,^21^ It also prevents muscle growth during development. The results of this study are the first to show a dose-dependent effect on human myoblast proliferation and regeneration capacity due to irradiation. These results indicate the occurrence of a significant change in myoblast proliferation and regeneration ability.

The myoblast stage of developing skeletal muscle is sensitive to ionizing radiation, and it is thus essential to devote particular attention to radiotherapy during infancy, while the sensitivity of adult skeletal muscle mass to ionizing radiation is still controversial. Most data suggest 6–11 that adult skeletal muscle is resistant to ionizing radiation unless higher doses of radiation are applied. However, higher doses of radiation can cause myopathies and, in most cases, induced muscle atrophy. Skeletal muscle in cancer patients is often exposed to ionizing radiation during radiotherapeutic treatment. One of the most probable mechanisms contributing to post radiation myopathy and muscle atrophy is the inability of muscle to regenerate. Muscle regeneration is a key process, responsible for maintaining the integrity of the muscle mass and function throughout life and particularly after muscle injury.^22^,^23^ It has been demonstrated that autocrine secretion of IL-6 plays an important role in the regulation of myoblast proliferation and differentiation.^24^ We observed an acute decrease in IL-6 secretion, which was more prominent when myoblasts were exposed to lower doses of irradiation. The less prominent decrease of IL-6 secretion in myoblasts exposed to higher doses of irradiation might be the result of IL-6 release from damaged cells, although we could not detect significantly increased levels of necrotic cell markers. In some *in vivo* and *in vitro* studies, it was shown that radiation increases cytokine IL-1 and IL-6 levels^23^,^25^; higher levels of cytokines might be due to their release from cells other than muscle. Similarly, although more robust, a decrease in constitutive IL-6 secretion was also observed in primary myoblast culture after exposure to high doses of glucorticoids or to environmental stress or organophosphates.^16^,^17^ Our results and the results of previous studies^16^,^17^ show high sensitivity of human myoblast to various chemical and physical factors. The mechanism of this myoblast response might involve transmission through highly conservative cytoprotective HSPs.^26^,^27^ In this connection, we observed increased levels of HSP 27 and HSP 70 as an acute myoblast response to irradiation; both HSPs are typically induced in cells when exposed to stress factors. In the light of the observed acute effects of irradiation, a long term effect might also be expected. In the long term, we observed a reduced proliferation capacity of myoblasts and subsequently also a higher rate of cell death, as detected by an increased level of LDH activity, which is a specific marker of cell damage and necrosis. These results are not unexpected, particularly due to the observed decreased level of secretion of IL-6 in myoblasts exposed to irradiation. IL-6 is a potent stimulator of myoblast proliferation and a decreased level of IL-6 secretion after irradiation might severely diminish autocrine and paracrine IL-6 activity.

In conclusion, our results show that myoblasts are sensitive to irradiation in terms of their proliferation capacity and capacity to secret IL-6. Since myoblast proliferation and differentiation are a key stage in muscle regeneration, this effect of irradiation needs to be taken into account, particularly in certain clinical conditions.
